# Tensor decomposition and machine learning for the detection of arteriovenous fistula stenosis: An initial evaluation

**DOI:** 10.1371/journal.pone.0286952

**Published:** 2023-07-25

**Authors:** Sepideh Poushpas, Pasha Normahani, Ilya Kisil, Ben Szubert, Danilo P. Mandic, Usman Jaffer

**Affiliations:** 1 Imperial Vascular Unit, Imperial College NHS Healthcare Trust, London, United Kingdom; 2 Department of Surgery and Cancer, Imperial College London, London, United Kingdom; 3 Electrical and Electronic Engineering Department, Imperial College London, London, United Kingdom; 4 Independent Researcher, United Kingdom; Jadavpur University, INDIA

## Abstract

Duplex ultrasound (DUS) is the most widely used method for surveillance of arteriovenous fistulae (AVF) created for dialysis. However, DUS is poor at predicting AVF outcomes and there is a need for novel methods that can more accurately evaluate multidirectional AVF flow. In this study we aimed to evaluate the feasibility of detecting AVF stenosis using a novel method combining tensor-decomposition of B-mode ultrasound cine loops (videos) of blood flow and machine learning classification. Classification of stenosis was based on the DUS assessment of blood flow volume, vessel diameter size, flow velocity, and spectral waveform features. Real-time B-mode cine loops of the arterial inflow, anastomosis, and venous outflow of the AVFs were analysed. Tensor decompositions were computed from both the ‘full-frame’ (whole-image) videos and ‘cropped’ videos (to include areas of blood flow only). The resulting output were labelled for the presence of stenosis, as per the DUS findings, and used as a set of features for classification using a Long Short-Term Memory (LSTM) neural network. A total of 61 out of 66 available videos were used for analysis. The whole-image classifier failed to beat random guessing, achieving a mean area under the receiver operating characteristics (AUROC) value of 0.49 (CI 0.48 to 0.50). In contrast, the ‘cropped’ video classifier performed better with a mean AUROC of 0.82 (CI 0.66 to 0.96), showing promising predictive power despite the small size of the dataset. The combined application of tensor decomposition and machine learning are promising for the detection of AVF stenosis and warrant further investigation.

## 1. Introduction

Haemodialysis (HD) is an effective long-term renal replacement therapy that provides a lifeline for more than 30,000 people in the United Kingdom (UK) with end stage renal disease (ESRD) [1]. Arteriovenous fistula (AVF; a surgical anastomosis between an artery and a vein) is the preferred choice of vascular access for long-term HD [2,3] as it is associated with longer access survival and lower complication rates as compared with catheters [4]. However, only 16% of patients initiate HD with AVFs due to a high primary failure rate [5]. AVF failure is an important cause of mortality, hospitalisation and excess healthcare cost [6–8]. AVF failure also occurs, most often due to progressive stenosis secondary to neointimal hyperplasia and fibrosis from consecutive cannulations [9]. The evaluation of AVF maturation and early detection of stenosis is important to allow for vascular interventions in order to maintain patency. Regular clinical examination and duplex ultrasound (DUS) assessments are non-invasive, inexpensive and are routinely used for AVF surveillance [2].

DUS examination allows for detection of areas with significant velocity change by using a combination of B-mode (grey scale ultrasound), colour and spectral Doppler techniques [10,11]. However, a number of studies have demonstrated that these measurements are poor at predicting AVF outcomes [12–14]. This may be related to the limitation of conventional Doppler techniques, which are angle dependent and therefore can only provide information when flow is parallel to the beam direction, with inadequate estimation of the orthogonal components. Hence, DUS is significantly limited by tortuosity and complex multidirectional flow, which is inherent to AVF [15].

Over recent years, various non-Doppler ultrasound approaches for flow estimation have been developed and are categorised as vector flow imaging (VFI) techniques [16]. Some VFI techniques are based on the tracking of speckle patterns on B-mode ultrasound caused by scattered ultrasound signals arising from moving blood cells. However, the interpretation of these complex patterns remains challenging and there is a need for the application of novel solutions to interpret the complex flow patterns associated with AVFs.

B-mode ultrasound cine loops of AVF blood flow would seem to fulfill the ‘four V’s of big data’ (volume, veracity, velocity and variety) and exhibit a large degree of structural richness. These characteristics can restrict the application of classical analysis on the underlying features due to its “flat-view”. However, when re-arranged in multi-dimensional structures (tensors), the same data often admit much more convenient and mathematically tractable ways of analysis. Through the use of multi-linear techniques and tensor decomposition (TD) data can be extrapolated providing highly informative multi-way data representation [17]. However, such methods to analyse data had not been very popular, due to high demand for storage and computational resources, until recent advances in computer science and computer manufacturing diminished prohibitive restrictions associated with tensor decompositions. Tensors can bring distant pieces of B mode data closer together through the use of an additional dimensions but in raw form may contain a considerable amount of repetitive and redundant information. Tensor decompositions can be used to more efficiently represent data by extracting latent components which better depict underlying processes and their nature [18–20].

In this study, we aimed to evaluate the feasibility of detecting AVF stenosis using a novel approach incorporating tensor-decomposition and machine learning classification for the analysis of B-mode ultrasound cine loops. We found that:

- Tensor decomposition was effective at extracting compressed representations of ultrasound cine loops
- Tensor decomposition components can be used to train a Long Short-Term Memory (LSTM) artificial neural network quickly and efficiently.
- When the ultrasound recordings were cropped to a relevant region of interest prior to tensor decomposition, the resulting components contain enough information to train an LSTM to effectively classify presence or absence of stenosis.

## 2. Material and methods

We analysed previously routinely collected B mode and Doppler data from the vascular laboratory of Hammersmith Hospital (Imperial College Healthcare NHS Trust, London) for routine AVF surveillance. The data were obtained using a standard of care clinical protocol; therefore, formal written consent and national ethics committee approval was not required. However, local institutional approval was obtained. Anonymised data was analysed from patients aged 18 years or over with ESRD with newly created (6-weeks post creation) or established AVFs. Demographics including age, gender, fistula type and comorbidities were recorded.

Patients had undergone DUS surveillance of their AVF as per local clinical protocol. All DUS examinations were performed with an Aixplorer ultrasound scanner (Supersonic Imagine, Aix-en- Provence, France), using a high frequency linear transducer (2–10 MHz). Normal protocol involved resting for 5-minutes in a seated position and then being scanned with one arm leaning on the couch with minimum pressure to avoid any deformation of the AVF. B-mode ultrasound as well as colour and spectral Doppler interrogation was performed at the arterial inflow, anastomosis, and venous outflow for each patient. Real-time B-mode cine loops of the fistula were recorded at the arterial inflow, anastomosis, and venous outflow for each patient.

### 2.1. DUS detection of stenosis

Classification of stenosis was based on the assessment of blood flow volume, vessel diameter size, flow velocity, and spectral waveform features. Pre-defined criteria were used for the detection of stenosis at the efferent vein (peak systolic velocity (PSV) < 50, PSV > 400 cm/s, or diameter < 3.5 mm), the anastomosis (PSV > 400 cm/s) and the afferent artery (PSV > 400 cm/s, or bi/triphasic pulsatile flow [21], or volume flow of < 300 ml/min [22].

### 2.2. Overview

**Fig 1:**
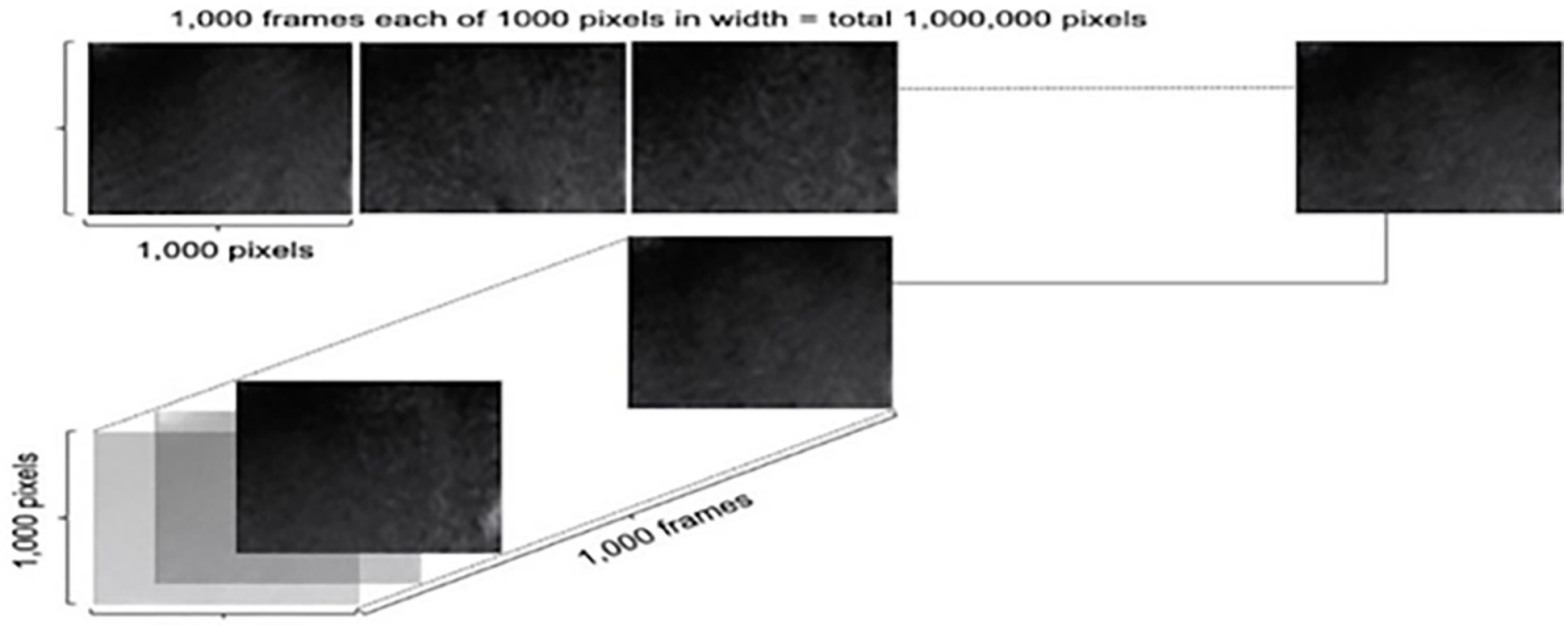
Video clip organised as “raw” multi–dimensional array.

B-mode video recordings were uploaded and reshaped into a tensor (N-dimensional array). The first 1,000 frames were used for all videos to capture at least one cardiac cycle. **Fig 1** shows consecutive frames from B-mode clips stacked into a tensor along “frame” dimension.

**Fig 2:**
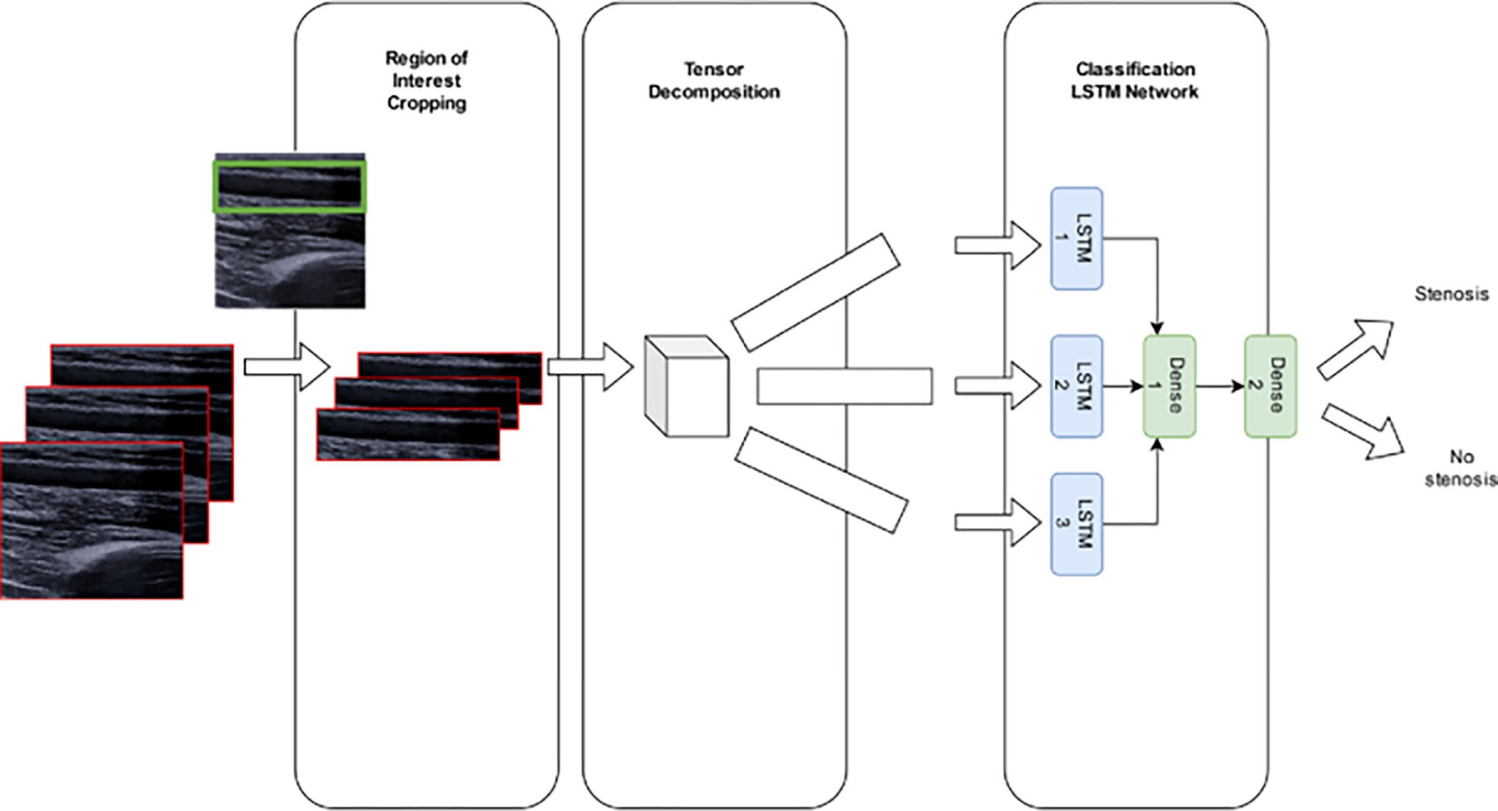
An overview of the proposed approach. Ultrasound video clips are cropped to regions of interest, then processed using tensor decomposition algorithms. The resulting three components from the decomposition are fed into a classifier neural network with three LSTM layers (one per input) followed by dense layers to predict presence or absence of stenosis.

An overview of our data processing pipeline and machine learning model can be seen in **Fig 2**. To start with, the raw data was manually cropped to just the region of interest to maximize the useful signal and remove pointless details by focusing on areas where the flow of blood was most visible. This step was initially skipped but was later found to be necessary to generate good quality features for the downstream machine learning model. Next, rather than attempting the expensive task of training a machine learning model directly on the cropped videos, we processed the cropped video clips using tensor decomposition to generate three components, each component consisting of a rank-1 tensor that contained useful extracted information from the video clips in a smaller form. These components highlight the important aspects of the data while dropping less useful aspects, allowing for quicker training of a machine learning model. The three resulting tensors were then used as inputs into a neural network utilizing multiple Long Short-Term Memory (LSTM) layers to learn to classify presence or absence of stenosis from the input sequences.

### 2.3. Feature extraction through tensor decomposition

**Fig 3:**
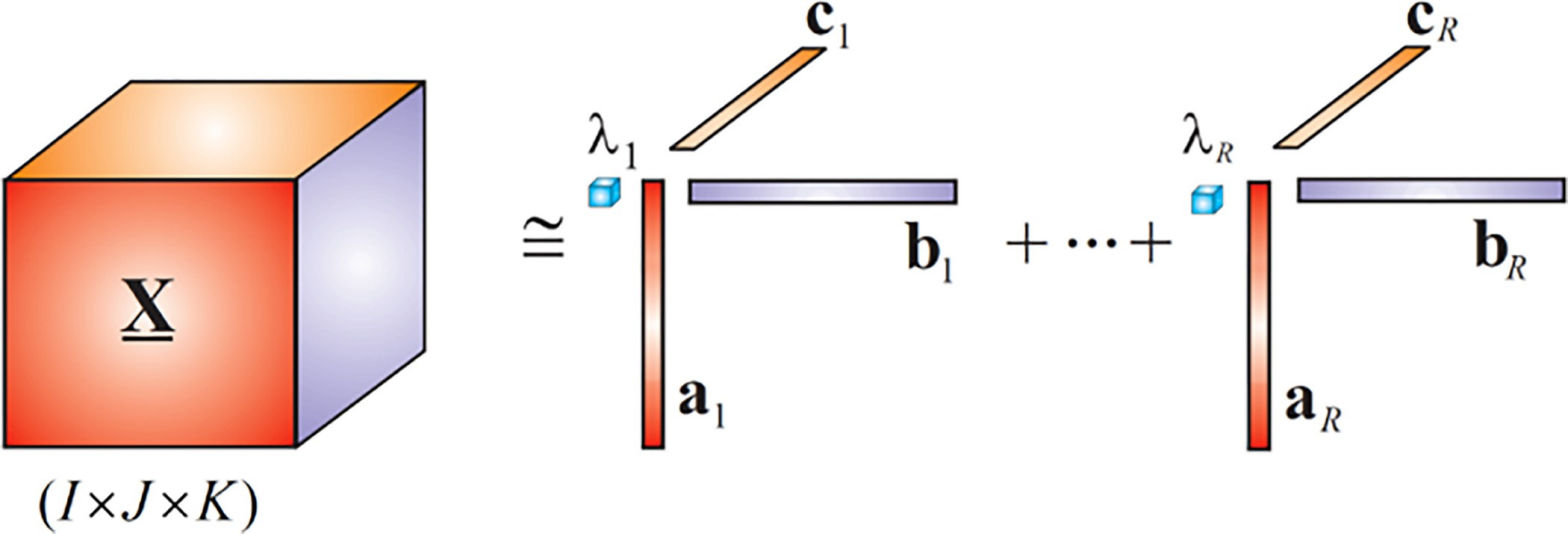
Canonical Polyadic Decomposition (CPD). An intuitive illustration of the way CPD decomposes the original “raw” data combined into a third–order tensor, resulting in a linear combination of spectral, temporal and spatial components which are independent from each other.

Tensor decompositions, using Canonical Polyadic Decomposition (CPD) and Tucker Decomposition (TKD), were applied. Tensor decomposition produced linear combination of components each of which is associated with a particular characteristic attributed to the “raw” data as illustrated in **Fig 3**.

In our experiments CPD was used to factorize a third order tensor $\underset{\_}{X}\in R^{I\times J\times K}$ into a sum of rank-1 component tensors.


$$\underset{\_}{X}≃\sum_{r=1}^{R}\lambda_{r}a_{r}\circ b_{r}\circ c_{r}$$ (1)

To find the components that best approximate *X* we need to find:

$$\min∥\underset{\_}{X}-[\underset{\_}{\Lambda };A,B,C]{∥}_{F}^{2}$$ (2)

The CPD was calculated using the alternating least squares method, which iteratively solves for one term at a time by fixing the other two. This process continues until convergence is reached, making the problem far more tractable.

The Tucker Decomposition of the same third order tensor $\underset{\_}{X}\in R^{I\times J\times K}$ is represented as a dense third order tensor *G* and a set of factor matrices.


$$\begin{array}{c} \underline{X}≃\sum_{q=1}^{Q}\sum_{r=1}^{R}\sum_{p=1}^{P}g_{qrp}a_{q}{}^{\circ}b_{r}{}^{\circ}c_{p}\\ \;=\underline{G}\times_{1}A\times_{2}B\times_{3}C \end{array}$$ (3)

This representation was computed using the truncated Higher Order Singular Value Decomposition (HOSVD) method, which computes each orthogonal factor matrix as the left singular matrices for every rank in tensor *X*.


$$\begin{array}{cc} X_{\left(1\right)} & =U_{1}\Sigma_{1}V_{1}^{T}\to A=U_{1}[1:R_{1}]\\ X_{\left(2\right)} & =U_{2}\Sigma_{2}V_{2}^{T}\to B=U_{2}[1:R_{2}]\\ X_{\left(3\right)} & =U_{3}\Sigma_{3}V_{3}^{T}\to C=U_{3}[1:R_{3}] \end{array}$$ (4)

After obtaining the factor matrices, *G* can be obtained as follows:

$$\underset{\_}{G}=\underset{¯}{X}\times_{1}A^{T}\times_{2}B^{T}\times_{3}C^{T}$$ (5)

Tensor decomposition was completed for both ‘full frame’ cine loops, which capture intraluminal blood flow as well as the surrounding tissue, and ‘cropped’ videos, which only captured intraluminal blood flow. After applying tensor decompositions, the original multi-dimensional array of a B-mode video recording was represented as a linear combination of independent components. The components characterise across horizontal and vertical axis of a video as well as its distribution across all frames of an original video. The latter component, i.e. frame feature, is of particular interest as it directly corresponds to speckle movements. Spectral analysis [23] was performed over the acquired signals to determine the power distribution of frequencies composing this frame component, as well as their evolution in time.

### 2.4. Machine learning model

Feature extraction was performed for every B mode cine video recording as described above. Each cine video was labelled with a binary label denoting presence or absence of stenosis as assessed using the standard DUS criteria. Mapping between labels and extracted components was used as an input for machine learning. As described, tensor decompositions were computed from both the ‘full-frame’ videos and the ‘cropped’ videos, and the performance of machine learning models trained on each were compared separately.

**Fig 4:**
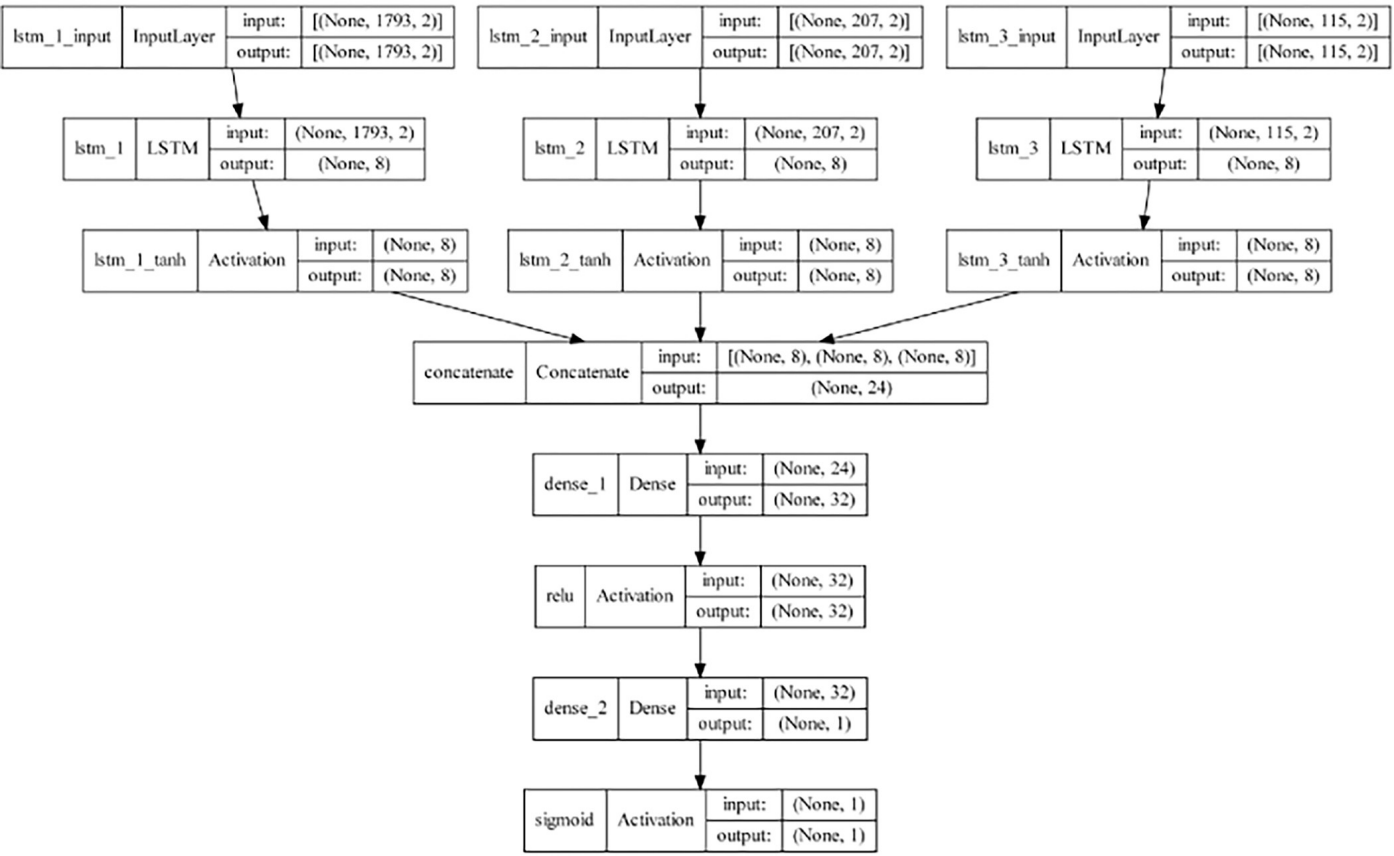
In–depth architecture of the multi–input LSTM neural network used to predict presence or absence of stenosis. Multi–input neural network with three inputs, the three components generated by the Tensor decomposition of the video: The frequency content, the time course, and the distribution over channels. Each input feeds into a separate LSTM layer before going through a hyperbolic tangent activation layer. The outputs are concatenated before being fed into a Dense layer followed by a ReLU activation layer, which then feeds into a single–neuron final Dense layer followed by a sigmoid activation for classification.

A multi-input neural network was used to predict the presence or absence of a stenosis on both the cropped and full-frame videos (**Fig 4**). The network consists of three separate Long Short-Term Memory (LSTM) layers with 8 cells that process each of the three components generated by the tensor decomposition process independently. These layers are specialized at dealing with sequences, such as those generated by the tensor decompositions. The LSTM outputs are passed through a hyperbolic tangent activation function and are then concatenated together and fed into a single Dense layer of 32 neurons, which is followed by a rectified linear unit (ReLU) activation before feeding into a single sigmoid-activated neuron which outputs the probability of stenosis.

The LSTM network was a natural choice due to its specialized handling of time-series data as a subclass of the Recurrent Neural Network (RNN). In recent times, the Transformer architecture has provided an alternative to RNNs with compelling results in machine translation tasks [24]. However, it has been noted that these Transformer networks are more liable to overfit their training data and can thus struggle to generalize [25]. Due to the small size of our dataset, overfitting was a very major concern–as a result, the older LSTM network was chosen over the newer Transformer networks for this proof-of-concept.

Prior to input to the neural network, data were Z-score normalized according to metrics obtained from the training set. The component sequences were also padded or truncated to be of uniform length per component based on the length of the longest sequence of that component in the training set. The network was trained using binary cross-entropy loss for a maximum of 100 epochs on mini-batches of size 1, with training being terminated early if validation loss failed to improve for five consecutive epochs. The loss function values were weighted by class, giving more weight to the rarer class, to counteract the effects of class imbalance [26]. The Adam optimizer was used with the default hyperparameters suggested in the paper: α = 0.001, β_1_ = 0.9, β_2_ = 0.999 and ε = 10^−8^ [26]. The weights of the LSTM layers were regularised during training using both the L1 and L2 regularisation penalties with a value of 0.01.

The dataset was divided into training, validation and testing sets to evaluate the model performance on unseen data and get a sense of its generalisability. The training set split contained 60% of the data, while the validation set and test set each contained 20%. The data splits were stratified both by video subject (arterial inflow, venous outflow, anastomosis) and by label to ensure even representation of the classes across the data splits. To get a better idea of how well the model generalises to new data and how much the performance varies based on different data samples, we generated multiple samples of the testing set using bootstrapping across 10,000 iterations, with each iteration drawing ‘n’ samples with replacements from the test set where ‘n’ is the total number of test data-points. The train, validation, and test split were stratified both by video subject (arterial inflow, venous outflow, anastomosis) and by label to ensure even representation of the classes across the data splits. The Area Under the Receiver Operating Characteristic (AUROC) curve of the model predictions was recorded for each iteration. The distribution of AUORC values for the whole-image classifier and the region-of-interest cropped classifier were compared using Student’s t-test, and the mean AUORC and corresponding confidence intervals for the mean were calculated for both.

All neural network models were trained without using a graphical processing unit (GPU)-acceleration on a server equipped with a Xeon E5-2630 v3 processer (2.40 GHz clock speed), owing to the effective summarisation of video features, the tensor decomposition algorithms in a lower-dimensional form.

## 3. Results

### 3.1. Demographics

Previously collected anonymised data from 22 patients were analysed (13 male, 9 female). Baseline characteristics for participants are included in Table 1. DUS measurements at the arterial inflow, anastomosis and venous outflow are presented together with resulting diagnosis of stenosis (**Table 2**). Video was not available for one patient as the fistula was completely occluded. Five videos could not be used due to poor image quality. A total of 61 out of 66 videos remained for analysis (anastomosis n = 19, arterial inflow n = 20, venous outflow n = 22).

**Table 1:** All baseline characteristics of patients whose scans were included.

	Stenosis or thrombosis?		
	N	Y	P
**n**	11	11	
**Gender = M (%)**	6 (54.5)	7 (63.6)	1.00
**Age (mean (SD))**	58.73 (14.19)	66.82 (12.15)	0.17
**AVF type (%)**			0.64
Distal BC	1 (9.1)	0 (0.0)	
Distal RC	0 (0.0)	1 (9.1)	
Prox BB	2 (18.2)	3 (27.3)	
Prox BC	6 (54.5)	6 (54.5)	
Prox RC	2 (18.2)	1 (9.1)	
**Diabetes = Y (%)**	5 (45.5)	6 (54.5)	1.00
**Hypertension = Y (%)**	7 (63.6)	5 (45.5)	0.67
**Smoking (%)**			0.21
Current smoker	3 (27.3)	1 (9.1)	
Non-smoker	5 (45.5)	3 (27.3)	
Present-smoker	3 (27.3)	7 (63.6)	
**Weight (Kg) (mean (SD))**	66.65 (9.83)	76.73 (12.93)	0.05
**BMI (mean (SD))**	24.38 (4.02)	27.35 (4.70)	0.13
**Pulse = Present (%)**	10 (90.9)	9 (81.8)	1.00
**Bruit (%)**			0.59
Absent	1 (9.1)	0 (0.0)	
Poor	3 (27.3)	3 (27.3)	
Strong	7 (63.6)	8 (72.7)	
**Thrill at anastomosis (%)**			0.49
Absent	1 (9.1)	0 (0.0)	
Strong	6 (54.5)	8 (72.7)	
Weak	4 (36.4)	3 (27.3)	
**Pain (%)**			0.32
Absent	6 (54.5)	9 (81.8)	
Mild	4 (36.4)	2 (18.2)	
Moderate	1 (9.1)	0 (0.0)	

SD, standard deviation; M, male; BC, brachiocephalic; RC, radiocephalic; BB, brachiobasilic; N, no; Y, yes; p, p–value.

**Table 2:** DUS measurements stratified by site of stenosis (arterial flow, venous flow, anastomosis).

	No stenosis	50–69%	70–99%	Occluded
n	13	3	4	2
**Venous**				
PSV (mean (SD))	188.6 (101.3)	516.0 (250.3)	498.0 (349.9)	NA
VF (mean (SD))	638.6 (375.3)	681.7 (362.7)	257.5 (126.5)	NA
Diameter maximum (venous) (mean (SD))	5.2 (3.6)	NaN (NA)	3.8 (2.2)	NA
Diameter minimum (venous) (mean (SD))	8.9 (7.7)	NaN (NA)	5.10 (5.1)	NA
**n**	**17**	**3**	**1**	**1**
**Anastomosis**				
PSV (mean (SD))	351.6 (173.1)	454.0 (99.3)	600.0 (NA)	NA
Diameter (mean (SD))	4.1 (1.8)	2.4 (0.6)	4.8 (NA)	NA
	**No stenosis**	**High resistance flow**		
**n**	**17**	**5**		
**Arterial**				
PSV (mean (SD))	189.1 (60.8)	221.0 (123.1)		
VF (mean (SD))	838.6 (459.8)	585.4 (699.2)		
Diameter (mean (SD))	5.3 (1.6)	4.7 (0.8)		

N, number; PSV, peak systolic velocity; VF, volume flow; NA, not applicable; SD, standard deviation.

### 3.2. Tensor decomposition analysis

**Fig 5:**
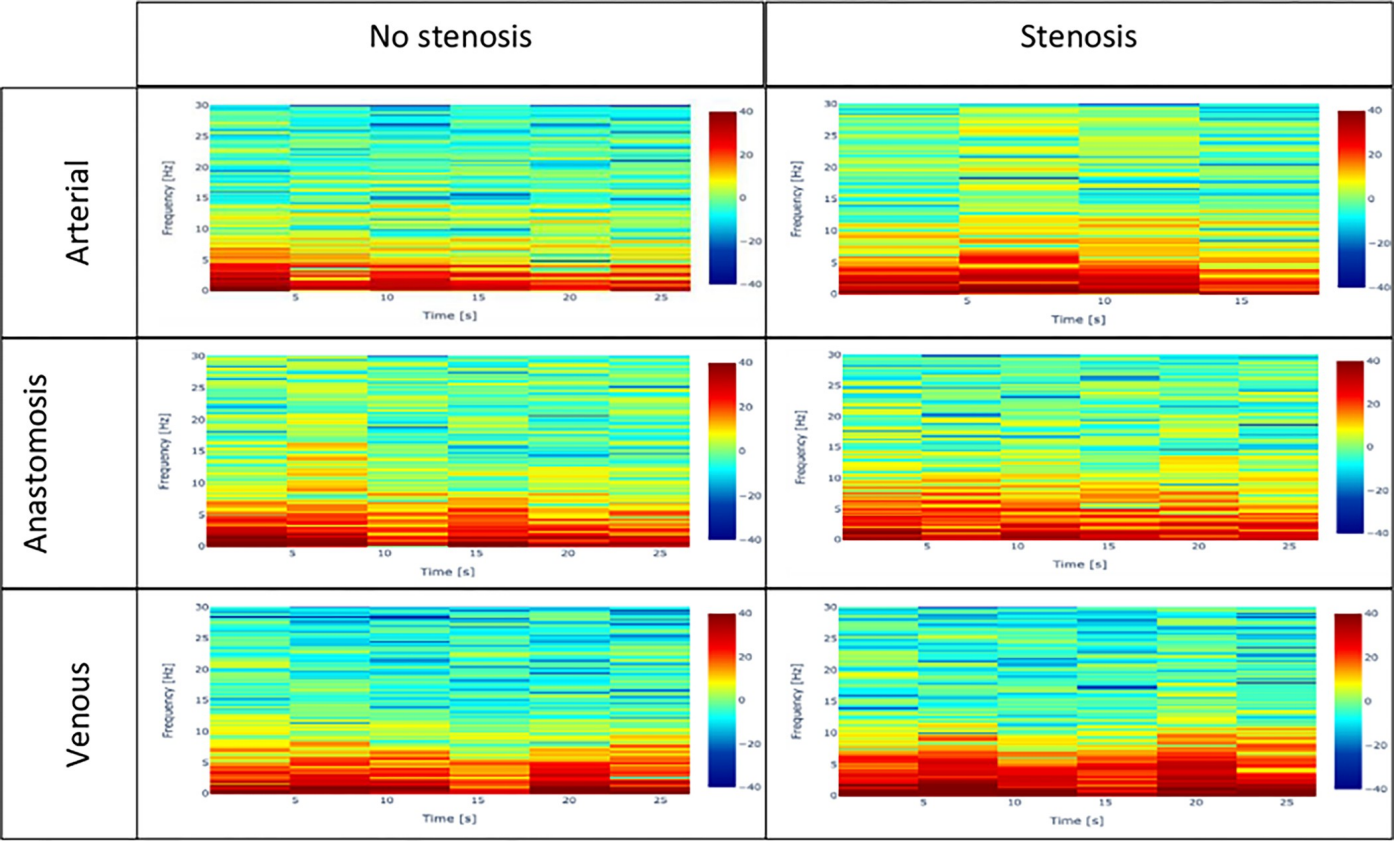
Spectrograms of arteriovenous flow (whole image) with and without stenosis. The spectrograms show the signals picked up in the whole image of arterial inflow, anastomosis, and venous outflow of fistulae with stenosis and no stenosis, which includes signals from the blood and the surrounding tissues. The Doppler readings in the vein with no stenosis are PSV (176 cm/s)–VF (475 ml/min)–diameter (2–5.8 mm) and with stenosis is diameter (<1 mm). In the anastomosis with no stenosis are PSV (386 cm/s)–diameter (4.4 mm) and with stenosis are PSV (500 cm/s)–diameter (2.4 mm). In the artery with no stenosis are PSV (187 cm/s)–VF (4950 ml/min)–diameter (5.8 mm) and with stenosis are PSV (315 cm/s)–VF (1800 ml/min)–diameter (5.2 mm).

**Fig 6:**
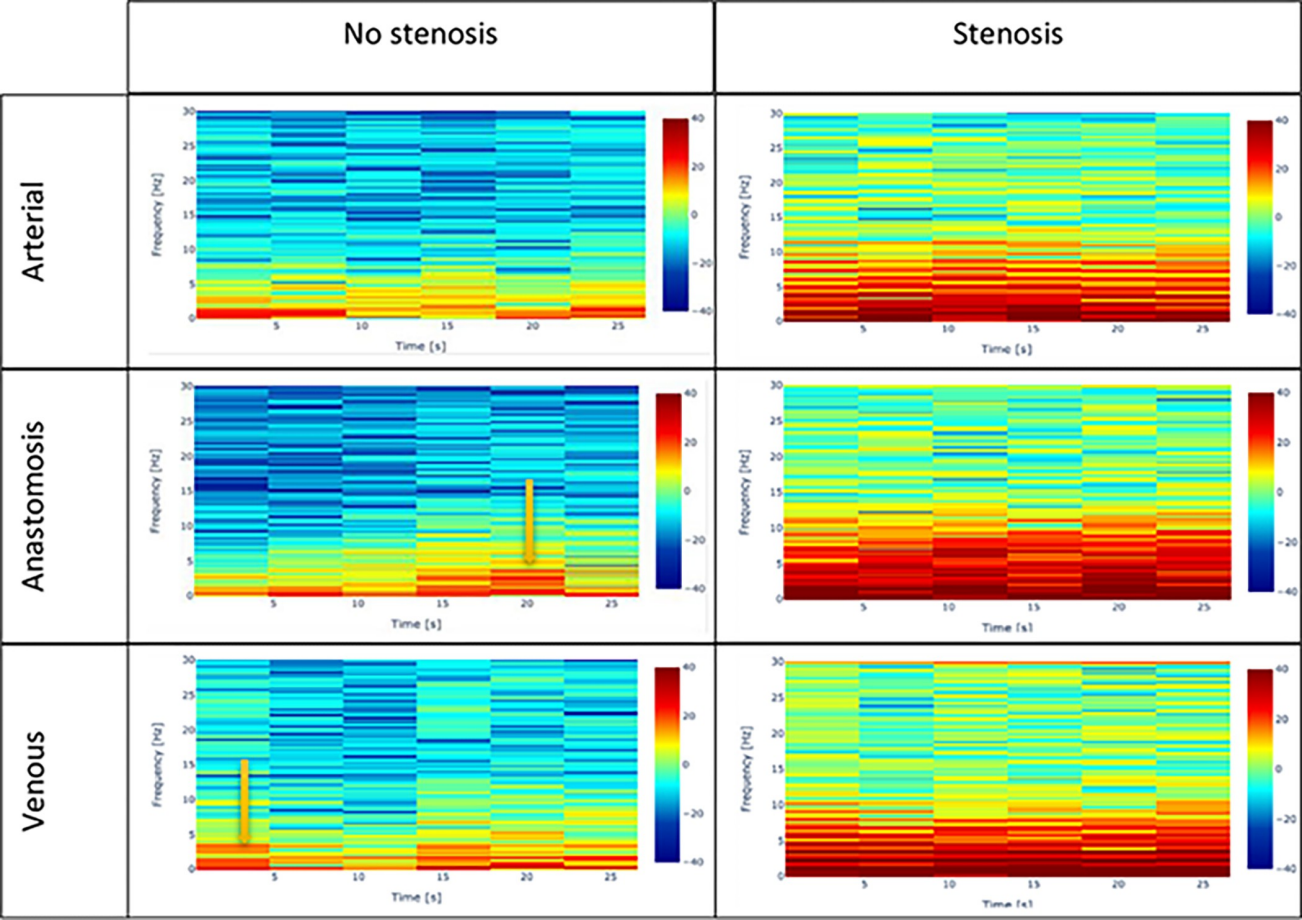
Spectrograms of blood flow only with and without stenosis. The spectrograms show the signals picked up in the blood flow only of arterial inflow, anastomosis, and venous outflow of fistulae with stenosis and no stenosis. The Doppler readings in the arterial with no stenosis are PSV (197 cm/s)–VF (1721 ml/min)–diameter (7 mm) and with stenosis are high resistive PSV (368 cm/s)–VF (290 ml/min)–diameter (5.6mm). In the anastomosis with no stenosis are HH 07 –PSV (250 cm/s)–diameter (3.2 mm) and with stenosis are PSV (340 cm/s)–diameter (2.9 cm)–stenosis (40–50%). In the venous with no stenosis are HH 04 –PSV (100 cm/s)–VF (610 ml/min)–diameter (4–9 mm) and with stenosis are PSV (693 cm/s)–VF (490 ml/min)–stenosis (45–50%). The *purple arrows* show the random peaks with high intensity, which correspond to an artefact either caused by the patient movement or by the operator moving transducer out of the plane.

On visual inspection, ‘Full frame’ videos, showed no consistent differences in spectrograms when comparing segments with and without stenosis (**Fig 5**). However, consistent differences in the spectrogram patterns were found (**Fig 6**) when comparing segments with and without stenosis for ‘cropped’ (blood flow only) videos. For these ‘cropped’ videos, higher frequencies across the entire sampling time were noted if stenosis was present on DUS criteria (**Fig 6**). Lower frequencies across the entire sampling time were seen more prominently on spectrograms of vessels with no stenosis which may correspond to laminar flow.

Also, artefacts in recording seemed to be identifiable as random peaks of high energies. These artefacts appeared similar to the patterns seen in the presence of stenosis. However, unlike spectrogram patterns from stenoses, these artefacts were fleeting (**Fig 6**).

### 3.3. Machine learning classification

**Fig 7:**
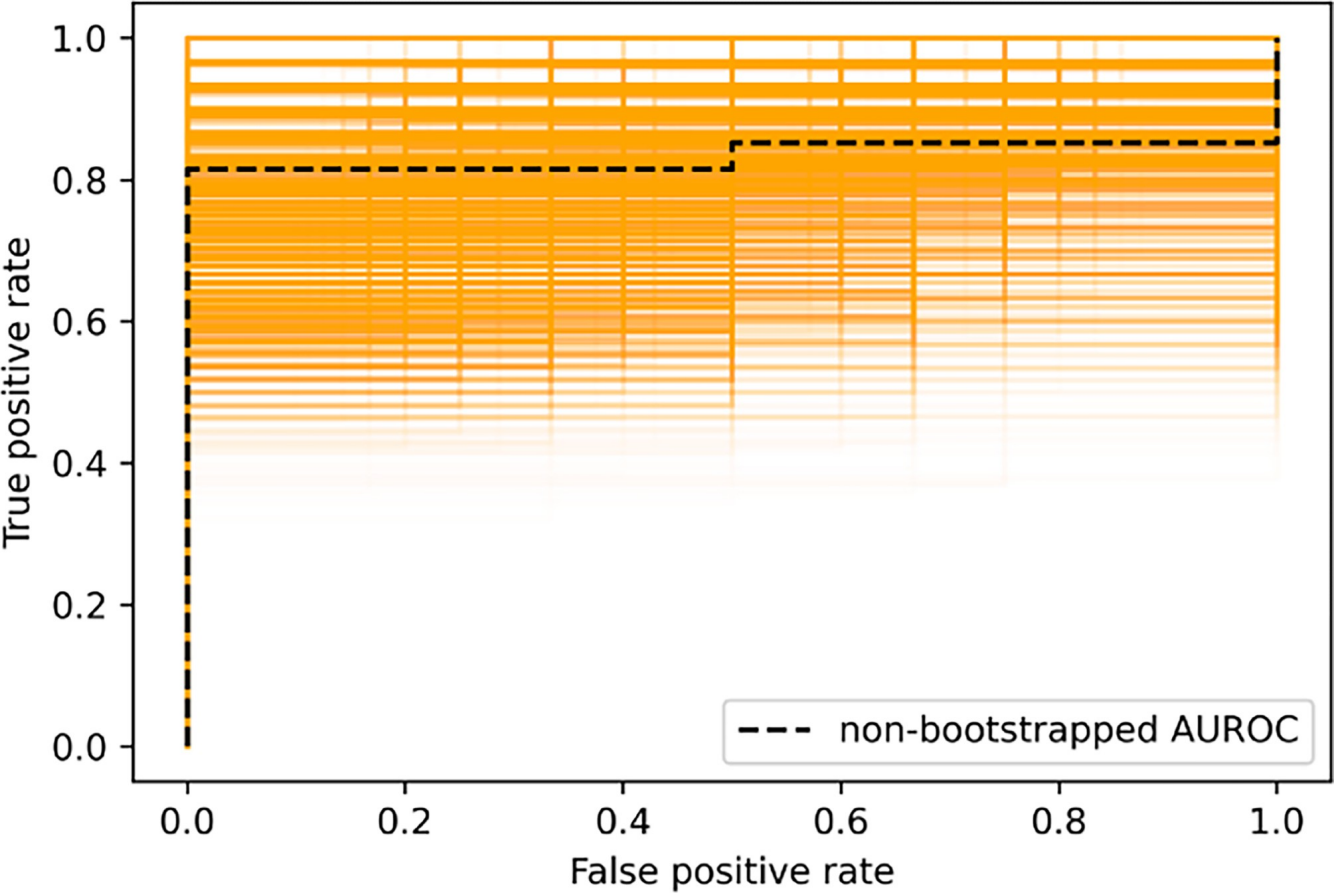
Bootstrapped ROC curves (n = 10,000) generated from the LSTM classifier predictions along with the ROC curve for the full test set. The non–bootstrapped AUROC was 0.821. The variation seen in the bootstrapped ROC curves shows the effects of a limited dataset size.

**Fig 8:**
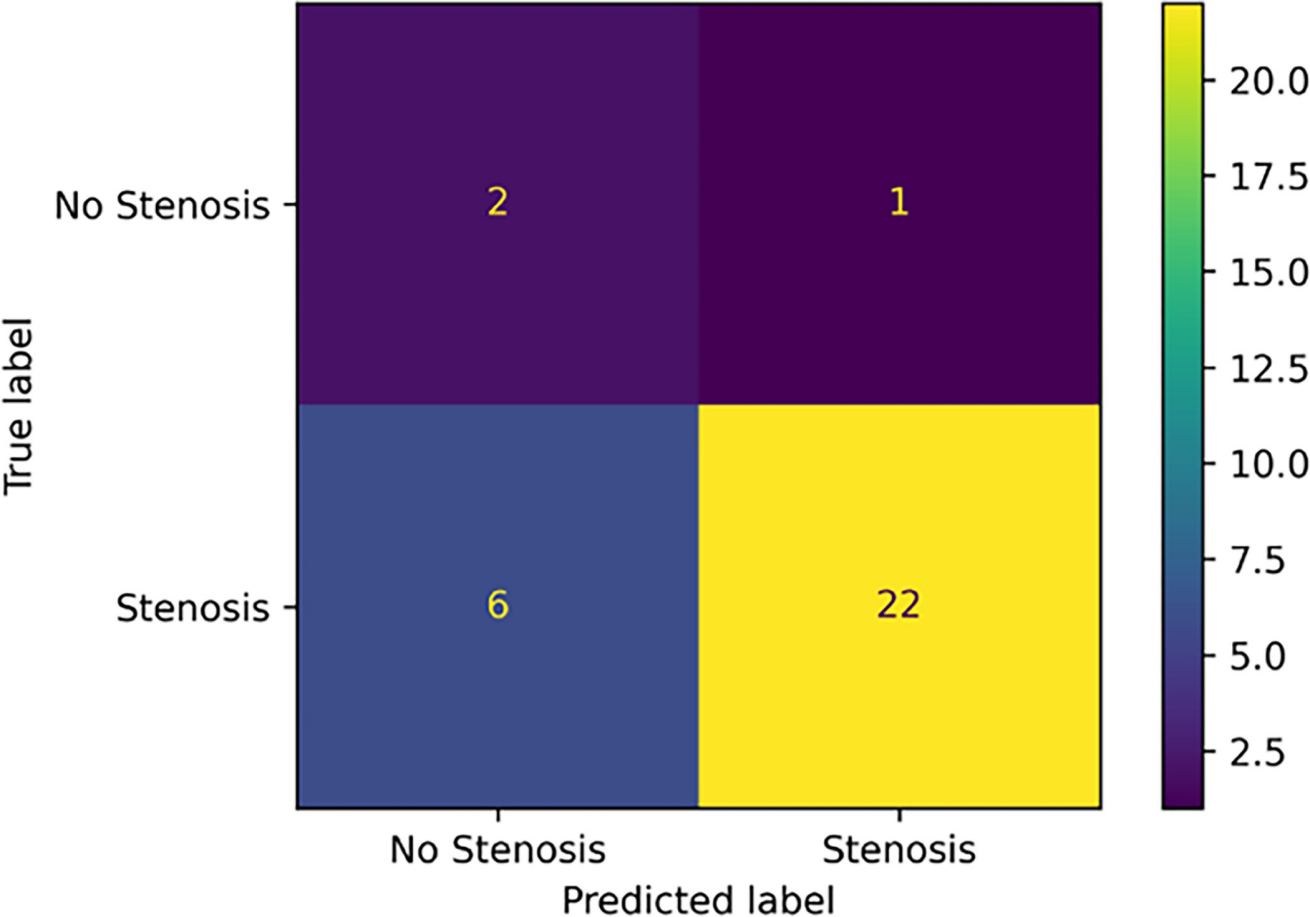
Confusion matrix comparing the model predictions against ground truths. The model is highly trustworthy when predicting a stenosis outcome, only mistaking one non–stenosis case for stenosis. Twenty–two stenosis cases are correctly classified as stenosis. Six stenosis cases are incorrectly classified as non–stenosis and two non–stenosis cases are incorrectly classified as stenosis.

The LSTM classifier trained on tensor decompositions obtained from ‘full frame’ videos failed to beat random guessing, achieving a mean AUROC value of 0.49 (CI 0.48 to 0.50). In contrast, the ‘cropped’ LSTM classifier performed better with a mean AUROC of 0.82 (CI 0.66 to 0.96) (**Fig 7**), showing promising predictive power despite the small size of the dataset, achieving a positive predictive value of 0.96 (**Fig 8**). The small size of the dataset may have also contributed to the relatively high degree of variance in model performance between runs, with the ‘cropped’ and ‘full frame’ classifier predictions having a standard deviation of 0.09 and 0.15 respectively. The difference between two distributions (cropped and full frame) is statistically significant (t-statistic 101.88, p <0.001).

## 4. Discussion

To our knowledge, this is the first feasibility study evaluating tensor-decomposition and machine learning for the detection of disturbed blood flow. We have demonstrated that training classification models, on features extracted through tensor decomposition of B-mode ultrasound videos of blood flow, show great promise at predicting AVF stenosis despite the small number of data-points. These results are particularly encouraging in the clinical scenario of AVF surveillance, which remains a challenging area where the application of novel solutions is necessary to improve clinical outcomes. DUS is a popular choice for AVF surveillance as it is non-invasive and cost-effective. However, its accuracy is limited due to the nature of DUS being operator and angle dependent [22]. Alternative non-Doppler technologies, such as the one evaluated in this study, may improve AVF patency by allowing for the early detection and treatment of stenosis, where necessary.

Comparison with prior attempts to solve this problem shows that out approach is competitive and takes a novel approach to the problem. Prior attempts to classify stenosis by applying an SVM to shunt murmurs resulted in low accuracy (55%) and underperformed human judges [27]. In contrast, our positive predictive value of 0.957 is slightly higher than the 0.917 achieved by a previous study of AVF classification using PPG signals, suggesting a potential use of this approach as a screening tool [28].

The technique described in this study has a number of notable and advantageous features. First, the tensor decomposition technique has proven to be effective at summarising useful classification features from B-mode videos of complex AVF blood flow. Secondly, summarising features in a lower-dimensional form has the added advantage of reducing computational requirements for training neural network models, allowing them to be trained without using a GPU. Additionally, this technique has the potential advantage of reducing the operator dependent nature of AVF DUS imaging as it is less dependent on ultrasound instrumentation and user interpretation.

However, there are a number of challenges to this technique that require further development and evaluation. Currently, this technique can be applied to B-mode videos of blood flow following image acquisition. However, for maximum clinical value and ease of use, real-time interpretation capability would be ideal. Furthermore, like VFI techniques, this technique also relies on frame by frame detection of speckle patterns. Therefore, further work is necessary to evaluate the optimal B-mode imaging settings for identifying speckle patterns scattered by red blood cells. Microbubble contrast agents enhance ultrasound signals from the blood [29] and have been used in a range of clinical applications [30]. These may improve the accuracy of this technique by enhancing the detection of speckles scattered from within blood by microbubbles.

Although, in this study we have applied this technique to AVF imaging, it may also prove useful in various cardiovascular ultrasound applications such as lower limb imaging and echocardiography.

## 5. Limitation

This study has a number of limitations. First, the results of this feasibility study are based on a small sample size. Typically, machine learning applications require large training data sets. However, despite a small training data set we were able to demonstrate some promising initial results. Future work should focus on gathering larger data sets to improve classification accuracy. Additionally, our cohort of patients represented a heterogenous group, with different types of newly formed and established AVF’s. Each group may have unique flow profiles that may have impacted on the accuracy of our classifier. Larger data sets may also help address this issue.

Finally, an important limitation of this study is that cine loops were classified according to the results of a reference DUS. We felt its use in this feasibility study was justified as it is a non-invasive and routine part of clinical evaluation at our institution. However, as discussed, this modality has a number of important limitations, which may impact on the accuracy of the results. Future work may consider using alternative imaging such as digital subtraction angiography, magnetic resonance angiography or clinical outcomes, such as AVF failure or patency. Future work should also evaluate the possibility of classifying stenosis severity.

## 6. Conclusion

Tensor decomposition extracts useful features from B-mode videos for the classification of AVF stenosis when the videos are cropped to regions of interest. These promising early results highlight the need for further development and evaluation as they suggest a far quicker and cheaper way of training artificial neural network models by using tensor decomposition components instead of training on the videos directly. This technique could potentially be used in a wide range of clinical applications including surveillance of AVF.

A natural improvement of the technique outlined in our experiments would be to automate the region of interest cropping. Since our focus in this work was to explore the possibility of cheap training on limited data and prove that the approach would work, we opted to manually annotate the regions of interest. The solution presented here would be far more practical if the region of interest cropping was automated, for example by training object detection models to automatically detect the relevant areas of the frame within a video.

In future work, it would be interesting to see whether Transformer networks might have a potential application to this problem given larger datasets with less danger of overfitting, given that Transformers have led the way in recent natural language processing tasks. Another area of interest to explore would be to see whether the tensor decomposition and classification models could be optimized together at the same time, rather than sequentially–end-to-end training may result in better performing models since the features extracted by the tensor decomposition could be guided by the overall optimization objective of the classification network, resulting in feature extraction that is more relevant to the classification task.

## Supporting information

S1 File (PDF)
