# Supplementary material for: Tensor decomposition and machine learning for the detection of arteriovenous fistula stenosis: An initial evaluation
Source: PLoS One. 2023 Jul 25;18(7):e0286952. doi: 10.1371/journal.pone.0286952 (PMC10368269; doi:10.1371/journal.pone.0286952)
Supplement: S1 File — (PDF) [file pone.0286952.s001.pdf]

I. Canonical Polyadic Decomposition

The Canonical Polyadic Decomposition (CPD) decomposes a tensor into a sum of rank-1 tensors. For example, given a 3-rd order tensor $\boldsymbol{\mathcal{X}} \in \mathbb{R}^{I_1 \times I_2 \times I_3}$, the CPD takes the form of

$$\begin{aligned}\boldsymbol{\mathcal{X}} &= \sum_{r=1}^{R} \lambda_r \mathbf{a}_r^{(1)} \circ \mathbf{a}_r^{(2)} \circ \mathbf{a}_r^{(3)} \\ &= \boldsymbol{\mathcal{D}} \times_1 \mathbf{A}^{(1)} \times_2 \mathbf{A}^{(2)} \times_3 \mathbf{A}^{(3)} \\ &= [\![\boldsymbol{\mathcal{D}}; \mathbf{A}^{(1)}, \mathbf{A}^{(2)}, \mathbf{A}^{(3)}]\!]\end{aligned} \tag{1}$$

where $\boldsymbol{\mathcal{D}}$ is a diagonal core, that is a tensor with only zero entries except at $\boldsymbol{\mathcal{D}}(r, r, r) = \lambda_r \neq 0$. The same tensor can be unfolded into matrices, in the CPD format, by

$$\begin{aligned}\mathcal{X}_{(1)} &= \mathbf{A}^{(1)}(\mathbf{A}^{(3)} \circledast \mathbf{B}^{(2)})^T \\ \mathcal{X}_{(2)} &= \mathbf{A}^{(2)}(\mathbf{A}^{(3)} \circledast \mathbf{A}^{(1)})^T \\ \mathcal{X}_{(3)} &= \mathbf{A}^{(3)}(\mathbf{A}^{(2)} \circledast \mathbf{A}^{(1)})^T\end{aligned} \tag{2}$$

An extension of the CPD to the N-th order is expressed by

$$\begin{aligned}\boldsymbol{\mathcal{X}} =& \boldsymbol{\mathcal{D}} \times_1 \mathbf{A}^{(1)} \times_2 \mathbf{A}^{(2)} \times_3 \cdots \times_N \mathbf{A}^{(N)} \\ =& [\![\boldsymbol{\mathcal{D}}; \mathbf{A}^{(1)}, \mathbf{A}^{(2)}, \dots, \mathbf{A}^{(N)}]\!]\end{aligned} \tag{3}$$

whereby the mode-n unfolding of the underlying tensor is found by

$$\begin{aligned}\mathcal{X}_{(n)} = \mathbf{A}^{(n)}\mathbf{D}(\mathbf{A}^{(N)} \circledast \cdots \circledast \mathbf{A}^{(n+1)} \circledast \\ \circledast \mathbf{A}^{(n-1)} \circledast \cdots \circledast \mathbf{A}^{(1)})^T\end{aligned} \tag{4}$$

where $\mathbf{D} \in \mathbb{R}^{R \times R}$ is a diagonal matrix with $\mathbf{D}(r, r) = \lambda_r \neq 0$. The CPD is obtained as the solution to

$$\underset{\boldsymbol{\mathcal{D}}, \mathbf{A}^{(1)}, \dots, \mathbf{A}^{(N)}}{\arg\min} \left\| \boldsymbol{\mathcal{X}} - [\![\boldsymbol{\mathcal{D}}; \mathbf{A}^{(1)}, \dots, \mathbf{A}^{(N)}]\!] \right\|_F \tag{5}$$

An Alternating Least Squares (ALS) approach is adopted to solve for $\mathbf{A}^{(n)}$, while keeping $\mathbf{A}^{(1)}, \dots, \mathbf{A}^{(n-1)}, \mathbf{A}^{(n+1)}, \dots, \mathbf{A}^{(N)}$ fixed. The algorithm optimizes each $\mathbf{A}^{(n)}$ in succession, starting from $\mathbf{A}^{(1)}$, and repeating the process until convergence.

II. Tucker Decomposition

The Tucker Decomposition (TKD) decomposes an original tensor into a core tensor transformed (i.e. multiplied) by a factor matrix along each mode. For a 3-rd order tensor $\boldsymbol{\mathcal{X}} \in \mathbb{R}^{I_1 \times I_2 \times I_3}$, the TKD is expressed as:

$$\begin{aligned}\boldsymbol{\mathcal{X}} &= \sum_{r_1}^{R_1} \sum_{r_2}^{R_2} \sum_{r_3}^{R_3} g_{r_1 r_2 r_3} \mathbf{a}_{r_1}^{(1)} \circ \mathbf{a}_{r_2}^{(2)} \circ \mathbf{a}_{r_3}^{(3)} \\ &= \boldsymbol{\mathcal{G}} \times_1 \mathbf{A}^{(1)} \times_2 \mathbf{A}^{(2)} \times_3 \mathbf{A}^{(3)} \\ &= [\![\boldsymbol{\mathcal{G}}; \mathbf{A}^{(1)}, \mathbf{A}^{(2)}, \mathbf{A}^{(3)}]\!]\end{aligned} \tag{6}$$

where $\boldsymbol{\mathcal{G}} \in \mathbb{R}^{R_1 \times R_2 \times R_3}$ and $\mathbf{A} \in \mathbb{R}^{I_1 \times R_1}, \mathbf{B} \in \mathbb{R}^{I_2 \times R_2}$, and $\mathbf{C} \in \mathbb{R}^{I_3 \times R_3}$. Even though the subspaces spanned by $\mathbf{A}, \mathbf{B}, \mathbf{C}$ are unique, the corresponding factor matrices generally are not, as for the unconstrained TKD they are rotation invariant. In order to be unique an orthogonality constraint on their columns must be imposed. Always considering a 3-rd order tensor, the matricized versions of (6) are:

$$\begin{aligned}\mathcal{X}_{(1)} &= \mathbf{A}^{(1)}\mathcal{G}_{(1)}(\mathbf{A}^{(3)} \otimes \mathbf{A}^{(2)})^T \\ \mathcal{X}_{(2)} &= \mathbf{A}^{(2)}\mathcal{G}_{(2)}(\mathbf{A}^{(3)} \otimes \mathbf{A}^{(1)})^T \\ \mathcal{X}_{(3)} &= \mathbf{A}^{(3)}\mathcal{G}_{(3)}(\mathbf{A}^{(2)} \otimes \mathbf{A}^{(1)})^T\end{aligned} \tag{7}$$

Moreover, the TKD can be readily extended to any N-th order tensor, in which case $\boldsymbol{\mathcal{X}}$ is decomposed as:

$$\begin{aligned}\boldsymbol{\mathcal{X}} =& \boldsymbol{\mathcal{G}} \times_1 \mathbf{A}^{(1)} \times_2 \mathbf{A}^{(2)} \times_3 \cdots \times_N \mathbf{A}^{(N)} \\ =& [\![\boldsymbol{\mathcal{G}}; \mathbf{A}^{(1)}, \mathbf{A}^{(2)}, \dots, \mathbf{A}^{(N)}]\!]\end{aligned} \tag{8}$$

and its mode-n unfolding takes the form of

$$\begin{aligned}\mathcal{X}_{(n)} = \mathbf{A}^{(n)}\mathcal{G}_{(n)}(\mathbf{A}^{(N)} \otimes \cdots \otimes \mathbf{A}^{(n+1)} \otimes \\ \otimes \mathbf{A}^{(n-1)} \otimes \cdots \otimes \mathbf{A}^{(1)})^T\end{aligned} \tag{9}$$

Notice the similarity with the CPD. The TKD can be thought of as a generalization of the CPD, in which the core is no longer diagonal, but has entries throughout. In fact, the CPD can equivalently be expressed by Equations (8), (9) in which $\boldsymbol{\mathcal{G}}$ and $\mathcal{G}_{(n)}$ are substituted by $\boldsymbol{\mathcal{D}}$ and $\mathcal{D}_{(n)}$, respectively.

Adopting an ALS approach, it solves the optimization problem

$$\underset{\boldsymbol{\mathcal{G}}, \mathbf{A}^{(1)}, \dots, \mathbf{A}^{(N)}}{\arg\min} \left\| \boldsymbol{\mathcal{X}} - [\![\boldsymbol{\mathcal{G}}; \mathbf{A}^{(1)}, \dots, \mathbf{A}^{(N)}]\!] \right\| \tag{10}$$

where $\boldsymbol{\mathcal{G}} \in \mathbb{R}^{R_1 \times \cdots \times R_N}$ and the matrices $\mathbf{A}^{(n)} \in \mathbb{R}^{I_n \times R_n}$ have the constraint of being columnwise orthogonal.
